# Chinese Adaptation and Psychometric Properties of the Child Version of the Cognitive Emotion Regulation Questionnaire

**DOI:** 10.1371/journal.pone.0150206

**Published:** 2016-02-29

**Authors:** Wen Liu, Liang Chen, Philip R. Blue

**Affiliations:** 1 College of Psychology, Liaoning Normal University, Dalian, Liaoning Province, China; 2 School of Marxism, University of Science and Technology Liaoning, Anshan, Liaoning Province, China; 3 Center for Brain and Cognitive Sciences and Department of Psychology, Peking University, Beijing, China; University of Vienna, School of Psychology, AUSTRIA

## Abstract

This study aimed to validate a Chinese’s adaption of the Cognitive Emotion Regulation Questionnaire for children (CERQ-Ck). This self-report instrument evaluates nine cognitive emotion regulation strategies that can be used by children after experiencing a negative life event. The CERQ-Ck was evaluated in a sample of 1403 elementary students between the ages of 9 and 11 by using cluster sampling. All the item-correlation coefficients for CERQ-Ck were above 0.30. The internal consistencies of the nine factors suggested moderate reliability (0.66 to 0.73). Confirmatory factor analysis (CFA) indicated that the current version had the same structure as the original instrument (Tucker–Lewis index = 0.912, comparative fit index = 0.922, root mean square error of approximation = 0.032, standardized root mean square residual = 0.044). A second-order factor and a third-order factor structure were also found. Test–retest correlations (0.53 to 0.70, *ps* < 0.01) over a period of 1 month, which ranged from acceptable to moderately strong were obtained from a random and stratified subsample of elementary students (*N* = 76). In addition, we analyzed convergent validity in relation to CERQ-Ck and the Chinese version of the Children’s Depression Inventory model dimensions with a subsample of 1083 elementary students. Multiple-group CFA confirmed the measurement invariance for both the male and female groups (ΔCFI < 0.01, ΔRMSEA < 0.015). Overall, results indicate that CERQ-Ck has similar psychometric properties to the original instrument as well as with adequate reliability and validity to investigate the nine cognitive emotion regulation strategies during late childhood developmental periods.

## Introduction

Years of research have illustrated that emotion regulation plays a prominent role in the psychological development and mental health of children [1–3]. Emotion regulation refers to the process by which a person attempts to reduce, maintain, or increase an emotion [4]. Thus, emotion regulation encompasses a wide range of conscious and unconscious physiological, behavioral, social, and cognitive processes. Certain strategies may be primarily implemented at the cognitive level, such as thinking of solutions to a problem. According to the process-oriented model, numerous strategies depend on a combination of cognition and behavior [5].

### Cognitive Emotion Regulation

From a theoretical point of view, researchers emphasize the affinity between the models of cognitive emotional regulation (CER) and cognitive coping [6]. Coping refers to the efforts of an individual to master demands (conditions of harm, threat, or challenge) that are appraised (or perceived) as exceeding or taxing his or her resources [6]. The traditional coping model [7] contains both cognitive (e.g., figuring out what to do) and behavioral (e.g., sharing one’s feelings with someone else) coping strategies. Measurement instruments designed according to this model provide data that reflect the combined effectiveness of these two strategies but not that of each separate strategy. This limitation is addressed in the CER model, in which cognitive factors are conceptualized and measured independently of behavioral factors [6]. Cognitive emotion regulation refers to the cognitive means of handling the intake of emotionally arousing stimulations [6,8]. Given the operative nature of CER, its model can be easily analyzed in a research setting [6,9]. In response to the absence of an adequate assessment directly examining CER strategies, Garnefski and colleagues developed the Cognitive Emotion Regulation Questionnaire (CERQ) [6]. From an application perspective, the outcomes obtained using CREQ by this group indicate that cognitive emotional regulation processes moderately affect the relationship between risks in people experiencing negative life events (e.g., myocardial infarction, chronic disease, or cancer) [10–12] and certain mental disorder symptoms (e.g., depressive symptoms) [13].

### Cognitive Emotion Regulation Questionnaire

CERQ was developed to assess Dutch secondary school students' CER strategies in relation to stressful life events [6]. To date, this measurement remains the only available scale that adopts a conceptually pure quantification of CER strategies. The questionnaire was previously developed using adolescent and adult samples; however, it had been validated recently using a sample of children aged 9 to 11 [8]. Identical to CERQ, the version of the CERQ for children (CERQ-k) evaluates nine conceptually separate CER strategies, Self-blame pertains to projecting blame onto oneself. Acceptance signifies accepting what one has experienced. Rumination implies reflecting on feelings and thoughts associated with a negative event. Positive refocusing involves embracing positive thoughts rather than fixating on a negative and stressful event. Refocus on planning indicates thinking about the best next step to take after experiencing a negative event. Positive reappraisal means considering the possible positive results or interpretations of a negative event. Putting into perspective entails comparing a negative event to other possible outcomes. Catastrophizing refers to making a negative event out to be worse than it actually was. Finally, Other-blame suggests blaming others for what one has experienced. In a broad sense, empirical studies using children and adult samples suggest that these nine dimensions are sufficiently intercorrelated that they can be grouped into adaptive and less adaptive CER strategies [6,9,14,15]. Adaptive strategies include Positive reappraisal, Refocus on planning, Positive refocusing, Acceptance, and Putting into perspective, whereas less adaptive strategies include Self-blame, Catastrophizing, Rumination, and Other-blame.

### Development of CERQ

The psychometric property examination of CERQ-k in Dutch children indicated that the values of Cronbach’s α values ranged from 0.67 to 0.79 for adaptive CER strategies and 0.67 to 0.79 (except for Acceptance, 0.62) for less adaptive CER strategies [8]. However, this previous study did not perform confirmatory factor analysis (CFA) to verify the hypotheses in the model and test–retest reliability to assess temporal stability. Other versions of CERQ have been developed in several researches, such as the French version (CERQ-F) using a sample of French young adults [14], the Chinese version (CERQ-C) using a sample of Chinese university students [16], the Spanish version (CERQ-S) using a Spanish sample [9], the Persian version (CERQ-P) using a sample of university students in Tabriz [17], and the Turkish version (CERQ-T) using a Turkish sample [15]. These versions of CERQ confirmed sufficient Cronbach’s α values, such as those ranging from 0.72 (Self-blame) and 0.83 (Catastrophizing) in CERQ-T to 0.76 (Self-blame and Putting into perspective) and 0.90 (Refocus on planning) in CERQ-C. Temporal stabilities suggested that these versions of CERQ (excluding CERQ-F and CERQ-P) were stable. For example, the test–retest reliability for the nine subscales ranged from 0.50 (Other-blame) to 0.70 (Self-blame) in CERQ-T. Confirmatory factor analysis (CFA) revealed positive support for this underlying first-order nine scale structure in these versions of CERQ (excluding CERQ-P). Meanwhile, a second-order factorial model was confirmed in CERQ-F and CERQ-S. As mentioned earlier, these two higher order factors encompass CER styles that have been categorized as adaptive and less adaptive CER strategies. Validity was also established in several studies, including a comparison of CERQ with other measures related to affective functioning, such as the Children’s Depression Inventory (CDI) [8] and the Positive and Negative Affect Scale (PANAS) [9]. Positive correlations were observed between less adaptive CER strategies and the self-reporting of depressive symptoms, anxiety, and negative affect; adaptive CER strategies were negatively correlated with depression symptoms and anxiety but positively correlated with positive affect [9]. Overall, these results indicated that adaptive strategies were predictive of improved psychological functioning, whereas less adaptive strategies were predictive of depressive symptoms. Criterion-related validity of CERQ-k was established with a comparison of the CERQ-k to the Children’s Depression Inventory, Fear Survey Schedule for Children, and the Non-productive Thoughts Questionnaire for Children [8]. With regard to CERQ-k, Self-blame and Catastrophizing predict depressive symptoms, worry, and fearfulness, whereas Positive refocusing and Refocus on planning predict the absence of depressive symptoms [8]. Due to a good reliability and validity of the Chinese version Children’s Depression Inventory (CDI-C) relative to other scales [18,19], we decided to use CDI-C to test the validity of the Chinese Child Version of the Cognitive Emotion Regulation Questionnaire (CERQ-Ck). One of the objectives of the present study is to revise the CERQ-Ck. According to the previous studies, the hypotheses were as follows.

1. As in the original form, CERQ-Ck will demonstrate a nine-factor structure.
2. CERQ-Ck will exhibit good internal consistency and test–retest reliability values.
3. To confirm construct validity, adaptive CER strategies will correlate negatively with depression symptoms, whereas less adaptive CER strategies will correlate positively with depression symptoms.

### Studies Using CERQ

Two studies have established that males and females adopt different coping strategies. Women are more likely to report the use of Rumination, Catastrophizing, and Positive refocusing than men [20]. In addition, females score higher on Rumination, Catastrophizing, and Putting into perspective, whereas males score higher on Other-blame [9]. These findings illustrate that women generally tend to focus more on their emotional experience, acknowledging and discussing emotions more openly and ruminating more on sadness than men [20]. To the best of our knowledge, no study has yet tested whether the underlying measurement structure of all versions of CERQ is gender invariant. Psychological constructs, such as CER, constitute latent variables that cannot be measured directly. Consequently, psychological measures function as indicators of the latent construct. To compare a latent construct meaningfully across different groups, each observed indicator must relate to the latent variable in the same manner across all groups. The gender invariance of CERQ-k is important because differences in CERQ-k scores should only reflect differences in CER strategies. Hence, CERQ-k differences should not relate to differences in response tendencies between gender groups. For example, if one group avoids the extreme answer categories of the rating scale whereas the other group prefers these extreme response categories, then comparing the CER strategies of these groups can lead to rash and inappropriate conclusions. Consequently, one of the objectives of the present study is to examine the measurement invariance of CERQ-Ck across genders. The hypotheses was that structural invariance will exist across gender groups.

Two cross-cultural studies that tested CER strategies have demonstrated that cultural variations play an important role in examining the relationship between CER strategies and psychopathological symptoms [9]. Such cultural influences can explain why cognitive strategies that attribute the causes of a stressful event to others (Other-blame) in collectivist cultures (e.g., Chinese) are associated with high levels of anxiety [16], although they are not evident in individualist cultures (e.g., Spanish and Dutch). Similarly, cognitive strategies (e.g., Positive reappraisal) that contribute to a reappraisal of a stressful event in terms that favor personal growth are only associated with low levels of anxiety and depression in individualist cultural models [9]. In collectivist models, however, highly impersonal emotional regulation strategies (e.g., Positive refocusing) are preferably used [9,16]. Therefore, the present research aims to further investigate the relationship between strategies and depression symptoms in Chinese children.

### Research Objective

From a developmental point of view, the relationship between the cognitive strategies of children and psychological outcomes is an important research area. As children grow older, their emotion regulation ability increases and shifts from mainly external, behavior-oriented emotion regulation strategies to internal, cognitive-based strategies [8,21]. When young children reach the age of 8 or 9, they have learned to regulate their emotions by means of cognition, thoughts about themselves, and their feelings toward others [22]. Thus, theoretically valid CER measures for children are necessary. Nevertheless, psychological assessment tools for measuring the CER strategies of children remain limited in China. To fill in this gap, the present study revised a Chinese version of CERQ-k and examined its factor structure and psychometric properties using a sample of Chinese children.

## Materials and Methods

### Participants

The study protocol was approved by the institutional review board of the College of Psychology of Liaoning Normal University (China). The parents of the students had to sign a parental consent form for their children to participate in the study.

A total sample of 1403 elementary students, which consisted of 667 females (47.5%) and 736 males (52.5%), was tested via cluster sampling. All participants were aged between 9 and 11 (mean age = 10.14, *SD* = 0.87) and were from four primary schools in Dalian (Liaoning Province, China). To confirm convergent validity, a subsample of 1083 elementary students completed CERQ-Ck and the Chinese version of CDI (CDI-C) [23]. To assess the test–retest reliability of CERQ-Ck, a subsample of 76 participants with different ages and genders (mean age = 9.87, *SD* = 0.87, 35 females and 41 males) from the total sample was asked to answer CERQ-Ck 1 month later. A total of 21 psychology graduate students assisted the teachers in charge of the class to ensure the smooth progress of the study. The students were provided with a comprehensive description of the research in their classrooms. All the respondents who volunteered to participate in the study were rewarded with a gift (e.g., pencil).

### Procedure

The adaptation procedures used in this study were selected based on the guidelines developed by the International Test Commission [24,25]. Considering the advantages and disadvantages of the forward-adaptation and the backward-adaptation methods [26], all 36 CERQ-k items of the original instrument were translated into Chinese by two functionally bilingual translators into Chinese. The result of this work was assembled in a draft that was administered to 15 Chinese speakers of Chinese to obtain feedback. A psychology professor (whose first language was Chinese) and a postgraduate student (whose first language was English) who were proficient in both languages blindly translated the items back into English. Afterward, the final Chinese and the English back-translated versions were compared by three graduate students. Based on the judgments of a psychology professor, three primary school teachers, 12 psychology graduate students and 30 elementary students, CERQ-Ck was developed by modifying 8 items to improve the comprehension of children. For example, the item “I think that worse things can happen” (Putting into perspective) was replaced with “I think that certain things that happen to me are better than others” because the former is difficult for Chinese children to understand.

First, the project leader, a psychology professor, trained the teachers and psychology students (research assistants) regarding their task assignments, including stating the implications of the present study, reading the scale instructions using Mandarin, issuing and recovering questionnaires, and answering the questions of students. Second, all 1403 elementary students completed CERQ-Ck, whereas a subsample of 1083 elementary students also answered CDI-C administration of the teachers for approximately 1 hour. Finally, self-assessments were used to follow up with 76 elementary students after 1 month.

### Measures

#### The Chinese Child Version of the Cognitive Emotion Regulation Questionnaire

CERQ-k contains 36 items that assess 9 CER strategies using a 5-point Likert-type scale (1 = almost never, 5 = almost always). Each subscale consists of 4 items that represent different CER strategies, namely, Self-blame (e.g., “I think that I am to blame”), Acceptance (e.g., “I think that I have to accept it”), Rumination (e.g., “I often think of what I am thinking and feeling about it”), Positive refocus (e.g., “I think of nicer things”), positive reappraisal (e.g., “I think about what would be the best for me to do”), Refocus on planning (e.g., “I think that I can learn from it”), Putting into perspective (e.g., “I think that worse things can happen”), Catastrophizing (e.g., “I often think that it’s much worse than what happens to others”), and Other-blame (e.g., “I think that others are to blame”). Adaptive CER strategies include Positive reappraisal, Refocus on planning, Positive refocusing, Acceptance, and Putting into perspective, whereas less CER adaptive strategies include Self-blame, Catastrophizing, Rumination, and Other-blame. High scores on each scale indicate a pronounced use of the corresponding strategy. CERQ-Ck was then developed. The accepted draft of CERQ-Ck was formatted in a style similar to that of the original English version [8]. The Cronbach’s α values of the nine scales in the present study were 0.70 (Self-blame), 0.66 (Acceptance), 0.70 (Rumination), 0.69 (Positive refocusing), 0.73 (Refocus on planning), 0.67 (Positive reappraisal), 0.72 (Putting into perspective), 0.72 (Catastrophizing), and 0.71 (Other-blame).

#### The Chinese version Children’s Depression Inventory

CDI-C [23] consists of 27 items, and each item comprises 3 options that evaluate differences in depressive symptoms among children and adolescents aged from 7 to 17. The items were scored from 0 to 2, and high scores indicated considerably severe depression. To satisfy the requirements of the Children’s Ethics Committee, the 9th item that assessed the notion of suicide was excluded. Thus, the 26-item total score was calculated using this formula: total points of 26 items + (total points of 26 items /26) [27]. In the present study, the Cronbach’s α value of CDI was 0.89.

### Statistical Analyses

All analyses were conducted using SPSS version 20.0, except for CFA and multigroup CFA, which were conducted using Mplus version 7.0. The missing values (0.275% in CERQ-Ck, 0.578% in CDI-C) were inserted through the series mean method. An itemmetric analysis for the CERQ-Ck was executed for the total sample. The corrected item-total correlations were calculated to examine item validity. For internal consistency, the Cronbach’s α values of the nine scales and test–retest reliability were computed for the total sample. The correlations between the scales of CERQ-Ck and the total scores of CDI-C were calculated.

A CFA with robust maximum likelihood estimation (MLR) was conducted to test the structure of CERQ-Ck [28]. The model fit was assessed against several frequently cused goodness-of-fit indices: the chi-squared goodness-of-fit statistic [29], the Tucker–Lewis Index (TLI) [30], the comparative fit index (CFI) [30], the root mean square error of approximation (RMSEA) [31], and the standardized root mean square residual (SRMR) [32]. A value of 0.90 or higher for TLI and CFI implies an acceptable fit: RMSEA values less than 0.08 indicate a moderate fit, whereas values higher than 0.08 signify a poor fit [33]. SRMR values below 0.08 are regarded as a good fit [32].

Finally, multigroup CFA was performed to test structural invariance across gender groups. According to the multivariate non-normal distributed data in our study, the present work used MLR as estimator. A series of goodness-of-fit indices was used for model testing and comparison because measurement invariance incurred nested models. The significant results from the Satorra–Bentler scaled *χ*^2^ difference indicated that the model with smaller *χ*^2^ exhibited a statistically better fit. To identify a significant decrease of fit between a series of progressive restricted models, ΔCFI (< 0.01) was suggested as the criterion [34]. In addition, we calculated the ΔRMSEA (< 0.015) of highly restrictive invariant models for samples larger than 300, as suggested by Chen [34].

## Results

### Itemmetric Analysis and Reliability

Means, *SD*, skewness, kurtosis, and Cronbach’s α were calculated (Table 1). According to Finney and Distefano [35], a univariate kurtosis of less than 7 and a skewness of less than 2 demonstrate sufficient normality. The kurtosis and skewness values of the nine scales ranged from –0.47 to 0.58 and –0.57 to 0.90, respectively, which indicated that the data were of normal distribution. In the total sample, the Cronbach’s α values of the nine scales ranged from 0.66 (Acceptance) to 0.73 (Refocus on planning). All scales were acceptably reliable. The test–retest correlations were statistically significant at 0.53 (Acceptance, *p* < 0.01) to 0.70 (Catastrophizing, *p* < 0.01), which indicated acceptable temporal stability. The corrected item-total correlations ranged from 0.39 (Item 6, Table 2) to 0.59 (Item 16, Table 2).

**Table 1:** *Mean*, *SD*, Skewness, Kurtosis, Cronbach’s α, and the test–retest reliability for scales of the CERQ-Ck.

Scale	*Mean*	*SD*	Skewness	Kurtosis	Cronbach’s α	Test-retest correlations
*SB*	10.08	3.26	0.46	0.21	0.70	0.67**
Acc	11.60	3.52	-0.04	-0.34	0.66	0.53**
Rum	13.13	3.57	-0.17	-0.47	0.70	0.65**
P-Ref	13.94	3.58	-0.39	-0.23	0.69	0.66**
R-Plan	14.50	3.43	-0.51	0.01	0.73	0.67**
P-Rea	14.37	3.46	-0.41	-0.24	0.67	0.62**
PP	12.80	3.40	-0.57	-0.10	0.72	0.60**
Cat	8.94	3.77	0.81	0.21	0.72	0.70**
OB	7.89	3.25	0.90	0.58	0.71	0.67**

SB = Self-blame; Acc = Acceptance; Rum = Rumination; P-Ref = Positive refocusing; R-Plan = Refocus on planning; P-Rea = Positive reappraisal; PP = Putting into perspective; Cat = Catastrophizing; OB = Other-blame.

**: *p* < 0.01.

**Table 2:** Corrected Item-Total Correlation and Factor Loadings for items of the CERQ-Ck.

Scale	#items	*r*	factor loadings
Self-blame	1	0.43	0.65***
	10	0.46	0.69***
	19	0.55	0.57***
	28	0.49	0.59***
Acceptance	2	0.40	0.58***
	11	0.47	0.60***
	20	0.49	0.66***
	29	0.42	0.63***
Rumination	3	0.45	0.51***
	12	0.53	0.60***
	21	0.44	0.69***
	30	0.52	0.62***
Positive refocusing	4	0.43	0.45***
	13	0.54	0.64***
	22	0.41	0.57***
	31	0.51	0.62***
Refocus on planning	5	0.49	0.56***
	14	0.55	0.66***
	23	0.51	0.56***
	32	0.54	0.66***
Positive reappraisal	6	0.39	0.49***
	15	0.44	0.72***
	24	0.52	0.46***
	33	0.48	0.71***
Putting into perspective	7	0.49	0.58***
	16	0.59	0.66***
	25	0.47	0.62***
	34	0.50	0.69***
Catastrophizing	8	0.52	0.50***
	17	0.55	0.58***
	26	0.49	0.66***
	35	0.48	0.60***
Other-blame	9	0.46	0.62***
	18	0.48	0.72***
	27	0.54	0.59***
	36	0.50	0.59***

*r*: corrected item-total correlation

***: *p* < 0.001.

### Relationship between CERQ-Ck Scales

Table 3 provides the correlation matrix of the nine CERQ-Ck scales. The correlations among the nine scales ranged from –0.23 (Positive reappraisal, Catastrophizing, and Other-blame) to 0.53 (Refocus on planning and Positive reappraisal) in the total sample, with a mean correlation coefficient of 0.22.

**Table 3:** Correlations among Scales of the CERQ-Ck and the CDI-C.

Scales	DS	P-Ref	R-Plan	P-Rea	PP	Acc	SB	Rum	Cat
P-Ref	-0.21**								
R-Plan	-0.14**	0.43**							
P-Rea	-0.26**	0.48**	0.53**						
PP	-0.11**	0.29**	0.45**	0.35**					
Acc	0.30**	0.04	0.07*	0.01	0.06*				
SB	0.33**	-0.02	0.14**	0.07**	0.05	0.36**			
Rum	0.08**	0.26**	0.35**	0.34**	0.20**	0.27**	0.25**		
Cat	0.49**	-0.14**	-0.08**	-0.23**	-0.02	0.37**	0.31**	0.18**	
OB	0.28**	-0.05*	-0.13**	-0.23**	-0.04	0.26**	0.07*	0.04	0.46**

DS: Depression symptoms; P-Ref = Positive refocusing; R-Plan = Refocus on planning; P-Rea = Positive reappraisal; PP = Putting into perspective; Acc = Acceptance; SB = Self-blame; Rum = Rumination; Cat = Catastrophizing; OB = Other-blame.

*: *p* < 0.05,

**: *p* < 0.01.

### Convergent Validity

Correlations were calculated between the nine scales of CERQ-Ck and the total score of CDI-C. The results are provided in Table 3. As expected, the total scores of CDI-C were correlated significantly with the nine scales of CERQ-Ck. Positive refocusing, Refocus on planning, Positive reappraisal, and Putting into perspective were negatively correlated with depression symptoms. By contrast, the other five scales were positively correlated with depression symptoms. In particular, Acceptance was positively correlated with depression symptoms. Consistent with other children samples [8], the findings indicated a significant correlation between the five scales (Self-blame, Positive refocusing, Refocus on planning, Catastrophizing, and Other-blame) of the original CERQ-Ck and the self-reported depressive symptoms, which supported a high degree of convergent validity for CERQ-Ck.

### Factor Structure of CERQ-Ck

According to Mardia and Foster [36], if the value of Mardia’s normalized multivariate kurtosis is greater than 3, then it indicates a nonnormal distribution. In our study, a value of 170.8 for Mardia’s normalized multivariate kurtosis indicated a nonnormal distribution. As recommended by Sass [28], MLR with continuous obvious indicators is more appropriate when data are multivariate nonnormal.

In the total sample, the nine first-order factors of CERQ-Ck (Model A) were examined through CFA (Table 4). The indices of Model A demonstrated that the model provided a good fit to the data, with both TLI and CFI above 0.90 and both RMSEA and SRMR considerably below 0.08. The findings supported the underlying nine-factor structure of the original structure proposed by Garnefski et al. [8]. The standardized factor loadings were all significant and ranged from 0.45 (Item 4 to Positive refocusing) to 0.72 (Item 15 to Positive reappraisal and Item 18 to Other-blame), with a mean loading of 0.61, which suggested that the items generally converged meaningfully to the scales.

**Table 4:** Primary Goodness-of-Fit and Comparative Indices for the Model of the CERQ-Ck and Tests of structural invariance for multigroup model across gender groups.

Model	Type	*χ²*	*df*	TLI	CFI	RMSEA	SRMR	*S-BΔχ²*	Δ*CFI*	*ΔRMSEA*
The total sample	Nine factors	1369.203*	558	0.912	0.922	0.032	0.044			
	Second-order factors	1967.165*	584	0.857	0.867	0.041	0.078			
	Third-order factors	1977.155*	586	0.857	0.867	0.041	0.077			
Multigroup comparisons										
CERQ-Ck	Ci	1952.750*	1116	0.910	0.920	0.033	0.050			
	Mi	1983.201*	1143	0.912	0.920	0.032	0.051	30.36	0.000	0.000
	Si	2001.894*	1170	0.915	0.921	0.032	0.051	15.84	0.001	0.000
	Ri	2121.004*	1206	0.909	0.913	0.033	0.054	117.95***	-0.008	0.001

CERQ-Ck: The Chinese Child adaption of the Cognitive Emotion Regulation Questionnaire. *S-BΔχ²*: the Satorra-Bentler scaled *χ²* different. Ci: Configural invariance; Mi: Metric invariance; Si: Scalar invariance; Ri: Residual invariance.

*: *p* < 0.05.

***: *p* < 0.001.

To test whether the distinction between adaptive and less adaptive strategies would fit the data, a second-order factorial model made of two higher order factors (Model B) was proposed. As in the previous CFA, each strategy was defined by its respective items; however, two second-order factors were added. The first factor was defined by the four strategies that were supposed to be adaptive: Positive reappraisal, Refocus on planning, Positive refocusing, and Putting into perspective. The second factor was defined by the five strategies that were supposed to be less adaptive in the present study: Acceptance, Self-blame, Rumination, Catastrophizing, and Other-blame. The fit indices of Model B are presented in Table 4. RMSEA and SRMR suggested that the latent and measurement models, respectively, were acceptable. However, TLI and CFA were not within the acceptable cutoff (0.90). A third-order factorial model (Model C) was also examined. The nine first-order factors that formed the two second-order factors (adaptive and maladaptive CER strategies) formed a third-order factors (CER strategies). The fit indices of Model C, which are similar to those of Model B, are presented in Table 4. Considering the fit indices of three models and comparative fit indices (ΔCFI = -0.055) between Model B with Model A, the overall results suggested that the nine-factor model was better than the higher-order factor models.

### Measurement Invariance

We used multigroup CFA with increasingly restricted specifications conducted on the nine-factor model to examine measurement invariance across gender groups. Four common models were classified under this category: configural, metric, scalar, and residual invariance. Configural invariance was tested only for the same specifications for both females and males. This model is the first step to establish measurement invariance because it serves as the comparison standard for subsequent tests. Metric invariance requires the corresponding factor loadings for gender groups to be equivalent. If metric invariance is satisfied, then the obtained ratings can be compared across groups and observed item differences can indicate group differences in the underlying latent construct. Scalar invariance assumes that the intercepts of items are equal across the CERQ-Ck of gender groups. It is the last model necessary to compare scores across groups. All additional tests are optional and may be theoretically meaningful in specific contexts. Residual invariance, which is the most restricted model, demandes equal error variances. Each model is nested in the previous model, and measurement tests become increasingly restrictive. Multigroup CFA that follows this approach is widely accepted as the most powerful and versatile means to test measurement invariance. Consequently, a model is only tested if the previous model has been determined to be equivalent across groups. The results are summarized in Table 4. These models exhibited good fit indices, but the Satorra–Bentler scaled *χ*^2^ test between the scalar invariance model and the residual invariance model was significant, which indicated that the imposition of constraints (equal error variances across groups) resulted in statistically significant reductions in the fit of the residual invariance model compared with that of the scalar invariance model. However, considering the other comparative fit indices (e.g., ΔCFI < 0.01, ΔRMSEA < 0.015), the overall results suggested that the differences in error variances for both males and females were within the range of statistical tolerance to support equivalence across gender groups. The previously discussed models addressed full measurement invariance because they assessed each given element (i.e., factor loadings, item intercept, residual variance) to be equal in all groups, which indicated that each observed indicator of CERQ-Ck related to the latent variable was the same across gender groups.

## Discussion

This research aimed to validate CERQ-Ck and provide information on the adaptation procedure and psychometric properties of this version using a large sample of Chinese elementary students. By the age of eight or nine, children’s ER strategies progress from behavioral strategies to more sophisticated CER strategies, such as positive reappraisal, the cognitive re-interpretation of emotion eliciting events [8]. These cognitive transitions in middle childhood may have profound implications for the psychological development of children. The developmental period immediately preceding puberty is especially important. The CER strategies that are developed and used with increasing frequency during puberty are predictive of the strategies used throughout adulthood [10]. Thus, carrying out the research using CERQ-Ck and establishing adaptive CER strategies during the middle childhood period may have important protective effects and could reduce the risk of adult onset psychopathology.

CERQ-k developed primarily for Dutch children have often been inappropriate for Chinese children because emotional display rules under the collectivism culture have resulted in a framework different from that of western children groups. Therefore, translation was conducted with an integration of forward and backward procedures, which was important to allow the assessment of the reliability and validity of culturally sensitive CERQ-k. The item-correlation test was used to decide whether responses to a given test should be included in the set that was being averaged. A small item-correlation coefficient provides empirical evidence that an item is not measuring the same construct measured by the other included items. A correlation value of over 0.30 indicates that the corresponding item generally correlates positively with the scale [37]. Given that all the item-correlation coefficients for CERQ-Ck were above 0.30, all items of CERQ-Ck should be retained.

As expected, the internal consistencies were similar to those noted in the original version of CERQ-k (0.62 to 0.79) [8], and the values of Cronbach’s α ranged from 0.66 to 0.73. Given the number of items (each subscale has four items) in each scale, regarding the reliability coefficients to be moderate to acceptable is reasonable. The test–retest data of CERQ-Ck established test–retest correlations that ranged from 0.53 to 0.70, which suggested that the CER strategies were a relatively stable construct.

The CFA results demonstrated an adequate fit, which suited the nine-factor model identified in the original version for children of this questionnaire [8]. The RMSEA, CFI, TLI, and SRMR indices also indicated a positive fit. CERQ-Ck was also explained by a second-order factor structure (the adaptive and less adaptive CER strategies), as had been found in the original version of CERQ [8], CERQ-F [14] and CERQ-S [9]. A third-order factor structure (CER strategies) was examined for the first time. Although the authors’ first conceptualization of cognitive strategies was two higher dimensions (i.e., adaptive and less adaptive), the nine-factor model was better than high-order models based on the present study. In addition, the correlation patterns observed between the scales of CERQ-Ck and the scores of CDI-C offered further evidence for the validity of the Chinese instrument. Significant correlations with depression symptoms were noted for all nine scales. These results are consistent with previous theories and empirical findings [8]. As expected, Positive refocusing, Refocus on planning, Positive reappraisal, and Putting into perspective were negatively correlated with depression symptoms. This finding suggested that adaptive strategies might increase functionality and relieve depression symptoms. By contrast, Self-blame, Rumination, Catastrophizing, and Other-blame were positively correlated with depressive symptoms. Therefore, people may have a high tendency to develop depression symptoms by using these strategies. However, Acceptance, as an adaptive strategy, was also positively correlated with self-reported depressive symptoms among Chinese elementary students in the anticipative directions. This result was also established in a Chinese college students sample [16] and other previous studies; for example, the Acceptance subscale exhibited significant positive correlations with depressive symptoms in a round of revision of CERQ-T [15] and in definitive involuntarily childless people [38]. One possible explanation for this finding is that although Acceptance has generally been regarded as a functional CER [6], it may not be adaptive in situations when the stressor can be changed [9,39]. Considering that the sample of the current study consisted of elementary students who were mostly recruited from upper-level elementary schools in a developed city, Acceptance items might have been appraised as resigning passively to a stressful event. Most notably, items such as “It just happened; there is nothing I can do about it” might have implied a sense of hopelessness. In general, Acceptance results are mixed across studies. Further investigation and, if necessary, a revision of this subscale may improve the psychometric properties of CERQ-Ck and enhance the understanding of the role of Acceptance as a CER strategy.

Ensuring that the constructs are organized in a system of psychological models is crucial to examine the structural equivalence of psychological constructs and research gender effects further. In the multigroup CFA of gender variation, we discovered evidence for the progressive incorporation of constraints into factor loadings, which implied structural equivalency for CERQ-Ck across gender groups. Testing the equivalency of the factor structure for both gender groups resulted in accepting the hypothesis of configural invariance of CERQ-Ck. That is, females and males use the same frame of reference when rating their CER strategies on CERQ-CK. Metric invariance signifies that an equal change in CER strategies causes an equal change in the CERQ-Ck of gender groups. In practice, metric invariance denotes that patterns of relationships between CERQ-Ck and adjustment (such as depression) can be meaningfully compared across gender groups. Scalar invariance indicates that CERQ-Ck scores are similar for gender groups if the groups demonstrate the same biases toward CER strategies (i.e., if the score on the latent variable is zero), which implies that CERQ-Ck scores can be meaningfully compared across gender groups. Finally, error variance invariance indicates that CERQ-Ck measures CER strategies with the same degree of measurement error across gender groups [40]. Overall, evidence for the progressive incorporation of constraints into factor loadings was found, which indicated the measurement invariance of gender for CERQ-Ck. This finding is important for future studies that will investigate gender differences in CER.

The results indicated that CERQ-Ck could provide a valid assessment of CER strategies among Chinese elementary students. The support for the CERQ-Ck’s acceptable psychometric properties in the present study has two important clinical implications. One implication is that this tool can be used to diagnose the cause of the mood disorders regarding the frequency of utilization of CER strategies. The increased use of self-blame, rumination, and catastrophizing has always been related to mood maladjustment [12]. The use of adaptive cognitive strategies such as putting into perspective, on the other side, was associated with children psychological well-being [8]. Another implication is that this scale may deserve the attention of children's parents to the importance of socialization factors of CER that are typically influenced in children's living environment. It is noteworthy that the parental role (e.g., parental responsiveness to children’s displays of emotion, parenting styles that are controlling or caring, parent emotional expression, and parent emotion regulation) have been documented to play a fundamental role in children’s developing ability to self-regulate their emotions [41].

One of the limitations of this study is probably the use of a sample that is not representative of all Chinese elementary students. Thus, extending the research through a combination of stratified sampling and cluster sampling should be considered to verify the validity of this study in Chinese elementary students with other characteristics. Another limitation of this study is the use of observed variables (i.e., depression scores) instead of latent variables to gauge convergent validity. This limitation can reduce the validity of CERQ-Ck. Finally, common method biases are inevitable because all the measures used in the current study were self-reported questionnaires. Therefore, discriminant validity should also be established in future studies. A 18-item shorter version of CERQ is available in other languages [42], thus developing an 18-item shorter version of CERQ-Ck may be another possible direction for future research. Overall, the evidence obtained from this study is a considerable step forward for both researchers and practitioners. Moreover, this new tool allows future studies to analyze CER within the Chinese culture.

## Supporting Information

S1 File (RAR)
