# Correction: Chinese Adaptation and Psychometric Properties of the Child Version of the Cognitive Emotion Regulation Questionnaire

**DOI:** 10.1371/journal.pone.0303727

**Published:** 2024-05-09

**Authors:** Wen Liu, Liang Chen, Philip R. Blue

The Supporting Information file containing the Chinese adaptation of the Cognitive Emotion Regulation Questionnaire (CERQ) for children, as developed in this article, was omitted from the publication. It can be viewed below.

## Supporting information

S1 Questionnaire Chinese version of the Cognitive Emotion Regulation Questionnaire (CERQ) for children. (DOCX)
