# Supplementary material for: Correction: Chinese Adaptation and Psychometric Properties of the Child Version of the Cognitive Emotion Regulation Questionnaire
Source: PLoS One. 2024 May 9;19(5):e0303727. doi: 10.1371/journal.pone.0303727 (PMC11081488; doi:10.1371/journal.pone.0303727)
Supplement: S1 Questionnaire — (DOCX) [file pone.0303727.s001.docx]

**编号：**

**小学生认知情绪调节问卷**

亲爱的同学：

你好！每个人面对**不愉快的经历时**,都有自己的反应方式。下面的问题,希望你表明当你**经历不高兴的事情时（例如：当你被老师批评，或者当你弄坏了一件喜欢的玩具，或者当你被同学欺负等等）**,你最通常的想法。你的回答将不会公开，仅作为我们研究用。你的参与对我们来说非常重要，谢谢你的参与！每题有5个等级，请根据过去两个月内自己的表现，选择最接近自己的等级。请仔细阅读每一条句子,然后在你认为能表明你通常想法的等级上**划圈**。**每道题只有一个选项，请不要多选或漏题，否则问卷作废。**

1. 从不 2.几乎不 3.有时 4.经常是 5.总是

例如：当我跟朋友吵架时，我认为都是别人的错................................1   **②**  3   4   5

**请认真填写以下信息：**

姓名： 性别： 年龄（周岁）： 年级：

是否本地人： 是否独生子女：

请想象一两件你遇到的不愉快的事情，然后开始回答下面的问题！

………………………………………………………………………………………………………

|  | **从不** | **几乎不** | **有时** | **经常是** | **总是** |
| --- | --- | --- | --- | --- | --- |
| 1. 我认为自己应该受到责备 | 1 | 2 | 3 | 4 | 5 |
| 2. 我会想我必须接受已经发生的事情 | 1 | 2 | 3 | 4 | 5 |
| 3. 我常回想起我经历过的事情的感受 | 1 | 2 | 3 | 4 | 5 |
| 4. 我会想我经历过的更好的事情 | 1 | 2 | 3 | 4 | 5 |
| 5. 我会想我怎样才能做到最好 | 1 | 2 | 3 | 4 | 5 |
| 6. 我会想我能从经历的事情中吸取教训 | 1 | 2 | 3 | 4 | 5 |
| 7. 我会想这不是最糟糕的事情 | 1 | 2 | 3 | 4 | 5 |
| 8. 我会想我经历的事情要比别人糟糕得多 | 1 | 2 | 3 | 4 | 5 |
| 9. 我认为别人应该受到责备 | 1 | 2 | 3 | 4 | 5 |
| 10. 我认为自己很愚蠢 | 1 | 2 | 3 | 4 | 5 |
| 11. 我对发生的事情感到无能为力 | 1 | 2 | 3 | 4 | 5 |
| 12. 我总是想起我对发生过的事情的感觉和想法 | 1 | 2 | 3 | 4 | 5 |
| 13. 我会想一些其他的愉快的事情 | 1 | 2 | 3 | 4 | 5 |
| 14. 我会想如何能解决这些问题 | 1 | 2 | 3 | 4 | 5 |
| 15. 我会想我经历的事能让我感到“更成熟、更聪明” | 1 | 2 | 3 | 4 | 5 |
| 16. 我会想别人会遇到比我这件事更糟糕的事情 | 1 | 2 | 3 | 4 | 5 |
| 17. 我反复地想我经历的事情有多么糟糕 | 1 | 2 | 3 | 4 | 5 |
| 18. 我认为其他人很愚蠢 | 1 | 2 | 3 | 4 | 5 |
| 19. 我认为都是自己的错 | 1 | 2 | 3 | 4 | 5 |
| 20. 我会想我不能改变经历的事情 | 1 | 2 | 3 | 4 | 5 |
| 21. 我想去弄明白为什么我会对经历的事情有这样的感觉 | 1 | 2 | 3 | 4 | 5 |
| 22. 我会想某些好事而不是所发生的事 | 1 | 2 | 3 | 4 | 5 |
| 23. 我会想怎样去改变这种情况 | 1 | 2 | 3 | 4 | 5 |
| 24. 我会想我经历的事情也有好的方面 | 1 | 2 | 3 | 4 | 5 |
| 25. 我会想我经历的事情会比其他可能发生的糟糕事情要好多了 | 1 | 2 | 3 | 4 | 5 |
| 26. 我一直在想这可能是发生在自己身上最糟糕的事情了 | 1 | 2 | 3 | 4 | 5 |
| 27. 我认为都是别人的错 | 1 | 2 | 3 | 4 | 5 |
| 28. 我认为事情都是由于自己的原因造成的 | 1 | 2 | 3 | 4 | 5 |
| 29. 我会想我什么都做不了 | 1 | 2 | 3 | 4 | 5 |
| 30. 我总能想起我对发生了的事情的感受 | 1 | 2 | 3 | 4 | 5 |
| 31. 我会想愉快的经历 | 1 | 2 | 3 | 4 | 5 |
| 32. 我会想我怎么样能做到最好 | 1 | 2 | 3 | 4 | 5 |
| 33. 我会想我经历的事情并不全是坏处 | 1 | 2 | 3 | 4 | 5 |
| 34. 我会想我经历的事不是世界上最糟糕的事 | 1 | 2 | 3 | 4 | 5 |
| 35. 我会想我经历的事情有多么可怕 | 1 | 2 | 3 | 4 | 5 |
| 36. 我会想我经历的事情是由于别人的原因引起的 | 1 | 2 | 3 | 4 | 5 |
|  | | | | | |

计分方式：

COMPUTE 自责= (R1 + R10 + R19 + R28)/4.

EXECUTE.
COMPUTE 接受= (R2 + R11 + R20 + R29)/4.
EXECUTE.
COMPUTE 沉思= (R3 + R12 + R21 + R30)/4.
EXECUTE.
COMPUTE 积极重新关注= (R4 + R13 + R22 + R31)/4.
EXECUTE.
COMPUTE 重新关注计划= (R5 + R14 + R23 + R32)/4.
EXECUTE.
COMPUTE 积极重新评价= (R6 + R15 + R24 + R33)/4.
EXECUTE.
COMPUTE 理性分析= (R7 + R16 + R25 + R34)/4.
EXECUTE.
COMPUTE 灾难化= (R8 + R17 + R26 + R35)/4.
EXECUTE.
COMPUTE 责难他人= (R9 + R18 + R27 + R36)/4.
EXECUTE.

COMPUTE 适应性策略=接受 + 积极重新关注 + 重新关注计划 + 积极重新评价 + 理性分析.

EXECUTE.
COMPUTE 不适应性策略=自责 + 沉思 + 灾难化 + 责难他人.
EXECUTE.
